# Advances in Retinal Tissue Engineering

**DOI:** 10.3390/ma5010108

**Published:** 2012-01-05

**Authors:** Matthew Trese, Caio V. Regatieri, Michael J. Young

**Affiliations:** 1 Department of Ophthalmology, Schepens Eye Research Institute, Harvard Medical School, Boston, MA 02114, USA; E-Mails: mtrese13@gmail.com (M.T.); caio.regatieri@schepens.harvard.edu (C.V.R.); 2 Department of Graduate Medical Sciences, Boston University, Boston, MA 02215, USA; 3 Department of Ophthalmology, Federal University of São Paulo, São Paulo 09210–170, Brazil

**Keywords:** retinal engineering, poly(lactic-co-glycolic acid) (PLGA), poly(lactic acid) (PLLA), poly( glycerol-sebacate) (PGS), poly(caprolactone) (PCL)

## Abstract

Retinal degenerations cause permanent visual loss and affect millions world-wide. Current treatment strategies, such as gene therapy and anti-angiogenic drugs, merely delay disease progression. Research is underway which aims to regenerate the diseased retina by transplanting a variety of cell types, including embryonic stem cells, fetal cells, progenitor cells and induced pluripotent stem cells. Initial retinal transplantation studies injected stem and progenitor cells into the vitreous or subretinal space with the hope that these donor cells would migrate to the site of retinal degeneration, integrate within the host retina and restore functional vision. Despite promising outcomes, these studies showed that the bolus injection technique gave rise to poorly localized tissue grafts. Subsequently, retinal tissue engineers have drawn upon the success of bone, cartilage and vasculature tissue engineering by employing a polymeric tissue engineering approach. This review will describe the evolution of retinal tissue engineering to date, with particular emphasis on the types of polymers that have routinely been used in recent investigations. Further, this review will show that the field of retinal tissue engineering will require new types of materials and fabrication techniques that optimize the survival, differentiation and delivery of retinal transplant cells.

## 1. Introduction

Degenerative retinal diseases are a large group of conditions that if left untreated can result in irreversible blindness. Two common retinal diseases are age related macular degeneration (AMD) and retinitis pigmentosa (RP). AMD is a complex multifactorial disease that is the leading cause of blindness in individuals over 60 [1]. It can be divided into two categories; neovascular (wet) or atrophic (dry). On the other hand, RP is a genetic disease that affects nearly 80,000 Americans [2]. These patients most commonly present with visual impairment in the first quarter of life, although there is significant variability from individual to individual [3]. Despite different etiologies, both of these degenerative retinal diseases are characterized by progressive photoreceptor cell death. Today there are a variety of treatment options for individuals with wet AMD, including the intraocular injection of anti-vascular endothelial growth factor (VEGF) drugs and photodynamic therapy. However, treatment options for individuals with atrophic retinal diseases, like dry AMD and RP, have been limited to dietary supplements and some preliminary drug trials, which are designed to delay disease progression [4]. Gene therapy is another potential therapeutic option that has gained support in recent years, primarily due to the success of a clinical trial that reprogrammed the mutant RPE65 gene in individuals with Leber congenital amaurosis [5]. The success of this study may have far reaching implications for people with atrophic macular degeneration. With nearly 3 million Americans affected with AMD and RP there is a pressing need to develop new treatment strategies to restore the vision to these patient populations [2].

In both atrophic AMD and RP, vision is lost because the photoreceptors die. The mammalian central nervous system (CNS) is limited in its ability to regenerate these dead or damaged neurons, consequently vision loss is irreversible. However, outer retinal death does not necessarily imply inner retinal death. In fact, recent post mortem studies of individuals with advanced disease states have shown that up to 88% of the retina’s inner nuclear layer and 48% of the ganglion cell layer remain viable despite significant outer retinal degeneration [6,7,8]. It is believed that the inner retina’s viability is a product of its dual blood supply, in which the outer retina is nourished by the fenestrated choriocapillaris and the inner retina is satiated by the retinal circulatory system [9,10]. This unique disease pathology provides a foothold for a variety of therapeutic options, including cell based therapies.

## 2. Candidates for Cell Therapy

In diseases such as AMD and RP, it is clear that areas of RPE atrophy are associated with degeneration of the adjacent photoreceptors [11]. Subsequently, two major strategies have emerged which aim to regenerate the degenerating retina. The first aims to restore the photoreceptors themselves, while the second attempts to replace the RPE. A number of cellular sources have been studied with the hope of finding an ideal donor cell. This list includes embryonic stem cells (ESCs), fetal tissue, progenitor cells, induced pluripotent stem (iPS) cells and adult tissue specific stem cells.

Embryonic stem cells are derived from the inner cell mass of the blastocyst [12,13]. These cells are characterized by their properties of unlimited self renewal and the ability to give rise to the body’s three germ layers (endoderm, mesoderm, ectoderm) [14,15] and therefore when given the appropriate cues, ESCs can potentially differentiate into any of the body’s over 200 cell types [16]. Driving ESCs towards a fully differentiated and post-mitotic RPE or photoreceptor cell fate *in vitro* has been performed by a number of groups [17,18]. However, post-transplantation complications have given rise to investigations using more mature progenitor cell retinal transplants.

Progenitor cells are isolated from developing tissue and exist in a more advanced otogenetic stage than ESCs. This means that progenitor cells are committed to the eye field and therefore progenitor cell differentiation potential is intrinsically more robust. Despite tremendous promise both ESC and progenitor cell research have been hindered by both ethical and immunologic concerns.

Induced pluripotent stem cells are adult somatic cells that have had pluripotent factors introduced into the adult genome, a process referred to as reprogramming [19]. Therefore, iPS cells represent an autologous pluripotent cell population that is widely available and mitigates the ethical concerns that have plagued embryonic stem cell research. However, altering donor cell DNA (which often includes the introduction of well-known oncogenes) carries a number of translational concerns, specifically tumorigenicity. Although an in-depth review of these cell types is beyond the scope of this paper, it is important to point out that these cells types have been the focus of retinal regeneration investigations because of their *in vitro* proliferative capacity, multipotent properties and differentiation potential.

## 3. Retinal Transplantation via Bolus Injection

Early studies using fetal tissue grafts demonstrated limited success and gave way to studies which injected boluses of donor cells or microaggregate suspensions into the intravitereal cavity or the subretinal space. In 2004, Haruta *et al.* showed that primate ESC derived RPE cells were capable of mature protein production (namely, RPE65, CRALBP and Mertk) and phagocytic activity, *in vitro* [20]. Next differentiated primate ESC-RPE cells were injected into the subretinal space of Royal College of Surgeon (RCS) rats, where it was observed that primate ESC-RPE were capable of survival and supported photoreceptor rescue as evidenced by the increased thickness of the outer nuclear layer (ONL) [20]. Further, this study and another using human ESC derived RPE showed that RCS rats who received a bolus injection of donor cells had significantly increased optokinetic head tracking times [20,21]. More recently, Vugler *et al.* showed that human ESC derived RPE were capable of mature protein production, shed outer segment phagocytosis and basal lamina production in both the *in vitro* and *in vivo* environment [22]. Perhaps most importantly, this study showed for the first time that the transplanted human ESC derived RPE mimicked gene expression *in vivo* as evidenced by the down regulation of the immature eye field marker Pax6 with the concurrent up regulation of mature RPE markers, like RPE65 [22].

On the other hand, retinal transplantations aimed at regenerating the sensory retina has largely been performed using neural and retinal progenitor cells. One of the first of these studies injected adult rat hippocampal derived stem cells into the vitreous of rats with degenerative retinal disease [23]. These progenitor cells demonstrated robust integration and the expression of neuronal markers, but they did not produce photoreceptor specific proteins [23]. Later, it was hypothesized that isolated multipotent neuroretinal stem cells, already committed to a retinal cell fate, would be more likely to differentiate into photoreceptors. More recent transplant studies have confirmed this hypothesis using mouse models. A number of authors have demonstrated that retinal progenitor and retinal precursor cells injected into the subretinal space are able to integrate within host retinas, differentiate and express photoreceptor specific proteins [24,25]. Further, this research suggested that the injected RPCs created functional synaptic connections within the host retina because mice that were exposed to light after RPC transplantation demonstrated moderate improvements in pupillary responses [25]. However, only a small fraction of donor cells were able to penetrate the outer retinal barrier, consequently functional improvements were limited.

Although these studies have demonstrated the potential of cell based therapies to regenerate the degenerating retina, they also showed that the bolus injection of stem and progenitor cells to the subretinal space resulted in disorganized and poorly localized grafts [26]. These outcomes are inherently related to the transplantation procedure and result in low rates of cell survival due to poor donor cell integration and injection reflux [26]. Injectable hydrogel cell delivery systems have gained popularity because they may limit donor cell death due to injection reflux [27]. Implanting a confluent sheet of donor cells has many appealing features which could aid in “rebuilding” the outer retina. For example, retinal sheet transplantation has the benefit of allowing the organized delivery of properly oriented cells to the areas of most severe retinal degeneration [28]. This is particularly important in RPE transplants where maintaining cellular polarity and differentiation has been correlated with the success of the graft outcomes [29]. However, the harvesting of primary tissue yet again raised ethical, availability and immunologic concerns. Subsequently, many of those interested in retinal transplantation have adopted a tissue engineering approach in order to address the issue of limited tissue availability. Previous investigations into bone, cartilage and vasculature have all demonstrated the ability of biodegradable polymers scaffolds to promote stem/progenitor cell’s ability to recapitulate complex tissues [30,31,32]. By using biocompatible polymers that slowly degrade, the donor cells were able to produce their own extracellular matrix and subsequently cell-polymer grafts successfully generated healthy donor tissue.

## 4. A Tissue Engineering Approach: Potential Polymers

With this construct in mind, the concept of an optimal polymer for retinal tissue engineering began to take shape nearly a decade ago. The ideal polymer must be biocompatible in the eye (generate little or no immune response in the subretinal space), but it must also be thin (<50 μm thick), porous (in order to allow diffusion from the choriocapillaris, the eye’s vascular coat), and biodegradable (it must slowly disintegrate through hydrolysis without altering retina’s extracellular milieu) [33]. Further, it should have a Young’s modulus similar to the delicate sensory retina; yet, robust enough to support surgical manipulation. A number of polymers meet many of these requirements and have been approved by the FDA for an array of applications, including drug delivery [34]. This list of polymers includes: poly(lactic-co-glycolic acid) (PLGA), poly(lactic acid) (PLLA), poly( glycerol-sebacate) (PGS), poly(caprolactone) (PCL) as well as many others [35,36]. One advantage of the polymeric retinal tissue engineering approach is that each polymer has different chemical properties that can be manipulated in order to meet certain specifications (*i.e.*, thickness, Young’s modulus, surface topographies, *etc*.). Manipulation of these variables provides a myriad of ways to optimize polymer based cellular delivery to the subretinal space.

In assessing the viability of this potential therapeutic strategy, investigators had to demonstrate that stem and progenitor cells were able to adhere and survive on these biodegradable polymers both *in vitro* and in the subretinal space. Early RPE studies cultured an established cell line (D407) on first generation copolymers made of PLLA and PLGA [37]. The D407 cell line is characterized by a robust proliferative capacity that, in this study was not hindered by a thin micropatterned PLGA composite polymer. In fact, after 7 days D407 cells had reached confluence, elaborated tight junctions and expressed the RPE’s iconic cobblestone morphology [37]. In an attempt to optimize this copolymer, a recent study fabricated 4 different polymers with 4 different ratios of PLLA and PLGA (10:90, 25:75, 50:50, 75:25 and 90:10) [38]. In each case cellular attachment and proliferation was achieved, however only the 25:75 PLLA-PLGA polymer was able to sustain cellular division and polymeric adherence for the course of the month long study [38]. Because the 25:75 PLLA-PLGA polymer was the thinnest and most porous polymer in the group, these findings suggest that the observed cellular viability resulted because of an optimization of these characteristics. Although immortalized cell lines display morphologic similarities to *in vivo* tissues, recent studies on tissue culture transwells have shown that ESC, iPS and fetal cell cultures more closely resemble actual RPE [39]. Transwell studies showed that all three primary cultures underwent melanization, attained high transepithelial resistance and mature protein expression [17,39,40]. These results have spurred investigations that have successfully cultured human embryonic stem cells on PLGA and parylene (a non-biodegradable polymer) [41,42,43]. These cells pigmented and produced mature RPE markers such as ZO-1, RPE65 and PEDF, bestophin and RDH5 on both substrates [41]. Further, human ESC-RPE seeded on parylene withstood surgical manipulation, maintained the appropriate morphology and survived for one month in the subretinal space of RCS rats [42,43].

Similarly, initial investigations focused on photoreceptor replacement also cultured multipotenet cells on PLLA-PLGA copolymers [26,44]. Lavik *et al.* showed that RPCs seeded on these copolymers, simultaneously down regulated immature “stemness” cell markers (Hes5, nestin, Hes1 and Pax6) and up regulated mature retinal markers such as glial fibrillary acidic protein (GFAP), however photoreceptor specific expression was not observed [44]. A similar study showed that when compared to bolus injection, PLLA-PLGA polymer cell delivery system increased cell survival 10 fold and increased the number of cells successfully delivered to the subretinal space by 16 fold [26]. Regardless of these successes, the copolymers used in these experiments were thick (≥150 microns), relatively inflexible and represented a significant barrier in the mouse subretinal space perhaps altering photoreceptor-RPE interactions [26].

Poly(glycerol-sebacate), or PGS, is another polymer that has been investigated as a scaffold for subretinal cellular delivery. PGS is a particularly appealing polymer for retinal transplantation because can be thin (45 μm), surface modified and has mechanical properties that facilitate surgical implantation. For example, a seeded PGS polymer graft can be scrolled, loaded into a syringe and after subretinal injection the polymer spontaneously unrolls exposing donor cells to the areas of desired integration [31]. PGS has proved to be suitable for progenitor cell culturing as indicated by high levels of survival, adherence and proliferation [33]. Similarly to PLGA copolymers, cells adherent to PGS elicited a down regulation of immature markers (Pax6, Hes1 and Sox2) [33]. Additionally, RPCs adherent to PGS scaffolds produced robust (>90%) calcium imaging responses in the presence of glutamate [33]. Together these *in vitro* results suggest that PGS alone induced mouse retinal progenitor cell differentiation and generated functional neurons [33]. When the seeded PGS grafts were placed in contact with retinal explants or surgically implanted into the subretinal space of C57bl/6 or rhodopsin knockout mice, there was an observable increase in the number of cells that migrated into the host retina [33].

Recent studies using poly(e-caprolactone), or PCL, as a substrate for retinal progenitor cell culture and transplantation have had encouraging results. The first PCL polymers were designed to incorporate both short (2.5 μm) and long (27 μm) nanowires projecting from a thin (5 μm) polymer base [45]. This construct was intended to allow a pathway for donor cells to migrate into the host retina and enhance cellular differentiation and integration. PCL, alone or with incorporated nanowires, represents the thinnest polymer thus far used in retinal transplantation. *In vitro* studies showed that mouse RPCs seeded on PCL adhered, proliferated and expressed lower levels of immature markers, like Sox2, Pax6 and Hes5, when compared to controls [45]. Additionally, this study showed that when compared to smooth polymers, those with incorporated nanowires promoted the migration of RPCs into retinal explants [45]. Once transplanted PCL seeded polymer scaffolds, demonstrated that RPCs were capable of migration and integration into the host retina [45].

## 5. Histologic Analysis of Subretinal Transplantation

**Figure 1:**
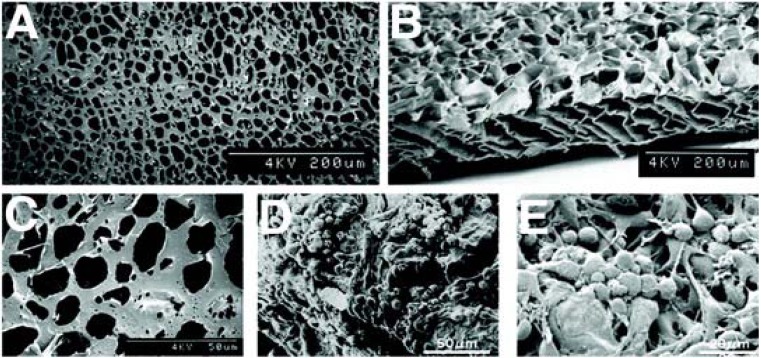

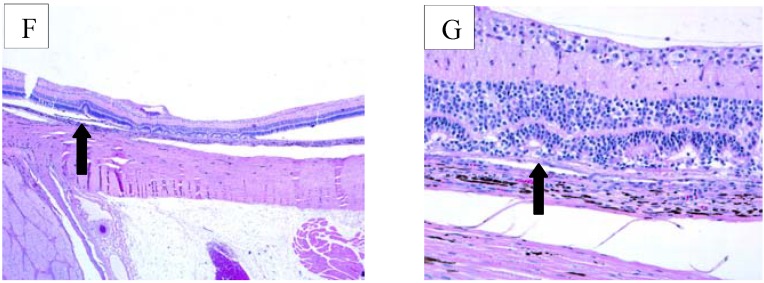
PLGA-PLLA Co-polymer. Scanning Electron Micrographs, adapted from Tomita *et al.* 2005 [26], depict the PLLA-PLGA copolymer before (**A**–**C**) and after RPC seeding (**D**,**E**). Below are representative examples of histology from transplant eyes 30 days after transplantation. The arrow of the left image (**F**) depicts the location of the polymer in the subretinal space. The arrow on the right (**G**) shows a disruption of the retinal layers due to an immune-like response.

All three of the previously mentioned candidate polymers were able to support stem/progenitor cell differentiation, survival and delivery. What separates these polymers and defines a front runner has been long term post transplantation histologic analysis. The standard of polymer investigations has been PLGA, alone or in combination with PLLA. Our group has recently shown that PLGA supported transplants led to the distortion of the outer retina and implied a poor prognosis for functional visual recovery (Figure 1). A possible explanation for this observation is that PLGA’s high molecular weight and 6–12 months bulk degradation time produced local decreases in pH. We feel that this change in the local environment may provide an opportunistic influx of immune cells, such as macrophages, and produces a histologic image that resembles a foreign body response.

**Figure 2:**
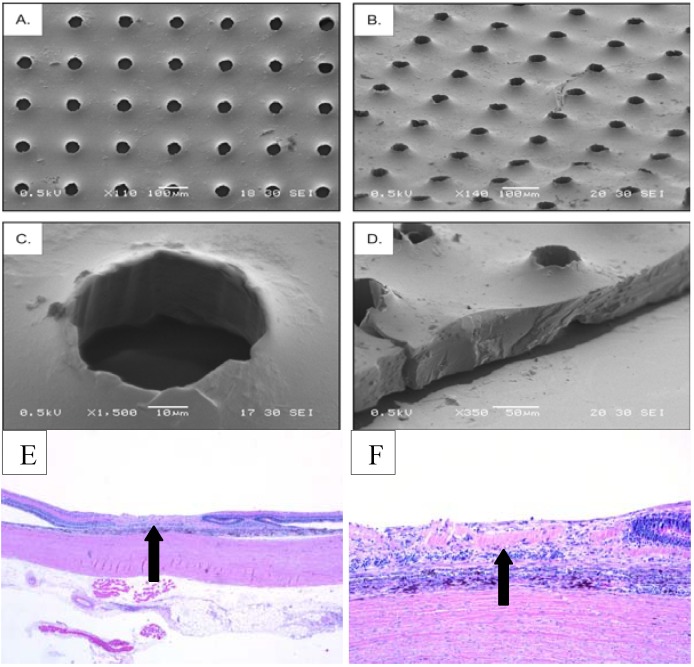
Porous PGS. Scanning electron micrographs, adapted from Neeley [46] depict a thinner polymer when compared to PLLA-PLGA. Further, the PGS polymer permitted surface modifications and scrollability. (**A**) and (**B**) show top views of the PGS polymer, while (**C**) and (**D**) display high magnification images of the polymer’s pores and edges, respectively. Despite improved fabrication techniques, the lower panel arrows depict the position of the polymer in the subretinal space (**E**) and the complete loss of the retinal layers 30 days post transplantation (**F**).

When compared to PLGA copolymers, PGS represented a significant step forward in terms of polymer fabrication. Perhaps most important was the decreased degradation time (30 days to 6 months) and surface hydrolysis of PGS which did not alter local pH. Additionally, PGS was thinner, more pliable and able to be surface modified. The specific surface modifications described by Redenti *et al.* were regularly spaced 50 μm diameter pores [33]. These pores were incorporated into the surface of the 40 μm thick polymer to shelter donor cells from the shear forces of transplantation [33]. Despite these advantages over PLGA, seeded PGS polymer transplants resulted in the complete loss of the outer retinal architecture as evidenced by the absence of the retinal layers (Figure 2).

**Figure 3:**
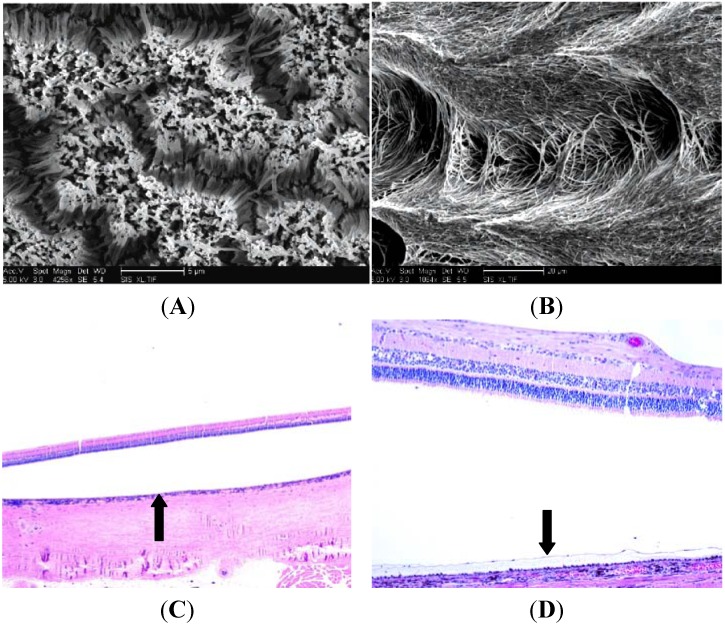
PCL with Incorporated Nanowire. Scanning electron micrographs show the PCL polymers with short (**A**) and long (**B**) incorporated nanowires [47]. This PCL polymer is the thinnest polymer to be used in retinal tissue engineering to date. In both the histologic images (**C**,**D**), the detachment of the neural retina from the RPE is an artifactual byproduct of the fixation process. The arrow on the left panel indicates the position on the polymer in the subretinal space. The inverted arrow on the right panel is demonstrating an example of a healthy retinal pigment epithelium, which further indicates the innocuous nature of the PCL polymer.

On the other hand, PCL scaffolds with short nanowires were capable of supporting donor cells and were well tolerated by the host retina. The ultra thin and porous profile of this polymer allowed for controlled 2–12 months surface degradation times and the passage of physiologically relevant factors [47]. Further, Figure 3 demonstrates the maintenance of the retinal architecture and the absence of an immune reaction. Although some of the underlying RPE was removed during surgery, the remaining RPE looks morphologically appropriate and none of the RPE has invaded the sensory retina. Importantly, the separation of neural retina from the RPE was a product of the fixation process. Finally, recent advances in polymer fabrication have allowed for variations of this PCL polymer to be constructed.

## 6. Limitations and Future Directions

These studies have shown that cell-substrate interactions play a pivotal role in cellular behavior and may prove to be an instrumental tool in driving stem/progenitor cells toward a differentiated cell fate. For example, Steedman *et al.* showed in an *in vitro* study that PCL scaffolds, lacking nanowires, but incorperating microtopographical cues effectively promoted the gene expression of the mature photoreceptor markers recoverin and rhodopsin [48]. As research into retinal tissue engineering progresses it is becoming clear that a single polymer will most likely not meet the needs of both RPE and photoreceptor replacement therapy. Previous studies have shown that donor RPE cells will not attach to or survive on an aged Bruch’s membrane [49]. This represents a significant hurdle that needs to be addressed in order for RPE replacement therapy to be an effective therapeutic option. One possible solution to this problem is the use of bioinert polymers that support RPE cell survival, functional differentiation and persist in the subretinal space. Conversely, photoreceptor replacement might be compromised by a permanent barrier in the subretinal space. Subsequently, a polymer that promotes stem/progenitor cell differentiation and dissolves without complication over a relatively short period of time might benefit photoreceptor replacement therapy. Because retinal regeneration must meet a diverse set of demands, it is imperative to continue to investigate other cell-polymer combinations which simultaneously differentiate stem and progenitor cells, facilitate cellular delivery and improve retinal transplantation techniques. To that end, one potential avenue of investigation would be the polymeric and simultaneous delivery of both RPE and photoreceptor precursors. Introducing these essential cellular layers of the outer retina, together, may prove to be a useful treatment option for individuals with late phase retinal degenerative disease.

Another potential complication of retinal transplantation is the possibility of an immune response. In animal models this response can be controlled with systemic immune suppression. However, systemic immune suppression can result in severe consequences especially in an elderly population, as would be the case for AMD patients. Previously, polymers have been designed to incorporate neurotrophic factors, matrix metalloproteases and other factors that slowly release their contents over time [50,51]. Similarly, loading a polymeric graft with an immunosuppressant like cyclosporine may potentially convey sustained and localized immune-suppression that would minimize immunologic concerns with regard to retinal transplantation. Another possibility that may alleviate concerns about graft rejection would be the use of induced pluiripotent stem cells. Because iPS cells could be taken from an autologous donor cell source, their ability to illicit an immune response may be greatly diminished. This notion, however, remains controversial because the complement system’s response to donor iPS cells has not been fully characterized [52].

Regardless of the cell source, technical challenges impede the translation of cell based therapies. The transplantation of undifferentiated cells has lead to concerns in regard to the tumorogenic potential of ESC, progenitors cells and iPS cells. Another potential issue for cell based therapy arises from the idea that stem cell manufacturing culture conditions may alter the gene and protein expression with which a patient’s immune system has been educated, thereby inciting an immune response [51]. Due to genetic reprogramming, iPS have their own concerns primarily in regard to incomplete reprogramming which may impact tumorgenicity and/or differentiation potential [53].

## 7. Final Remarks

Bolus injection retinal transplantation studies have laid the groundwork and provided the proof of concept that donor stem and progenitor cells can promote the functional recovery of vision in animal models of outer retinal degeneration. These studies also highlighted the need for more advanced cell delivery systems that optimize donor cell survival, differentiation and integration. The subretinal transplantation of stem and progenitor cells adherent to biodegradable polymers has emerged as a potential therapeutic strategy that has been shown to address many of these issues. This review was intended to describe how retinal degenerations can potentially be interrupted and reversed by cell based therapies. Further, this article was designed to inform the reader of the progress of retinal tissue engineering to date and provide examples of how this field is evolving. Although polymeric cellular delivery to the subretinal space holds a tremendous amount of potential, its clinical application remains on the horizon. In order for this potential therapy to become a success, engineers, biologists and clinicians must work together to develop new cell-polymer constructs and surgical techniques that are safe, effective and most importantly promote the functional recovery of vision.
